# Temporary Balloon Occlusion of the Common Hepatic Artery for Yttrium-90 Glass Microspheres Administration in a Patient with Hepatocellular Cancer and Renal Insufficiency

**DOI:** 10.1155/2013/560758

**Published:** 2013-04-27

**Authors:** Justin Smith, Ravi Murthy, Amit Lahoti, Bruno Odisio, Rony Avritscher, Beth Chasen, Armeen Mahvash

**Affiliations:** ^1^Department of Diagnostic and Interventional Imaging, UT Houston Medical School at Houston, 6431 Fannin Street, Houston, TX 77030, USA; ^2^Department of Diagnostic Radiology, The University of Texas MD Anderson Cancer Center, 1515 Holcombe Boulevard, Unit 1471, Houston, TX 77030, USA; ^3^Department of Internal Medicine, The University of Texas MD Anderson Cancer Center, 1515 Holcombe Boulevard, Unit 1471, Houston, TX 77030, USA; ^4^Department of Nuclear Medicine, The University of Texas MD Anderson Cancer Center, 1515 Holcombe Boulevard, Unit 1471, Houston, TX 77030, USA

## Abstract

The most severe complication of yttrium-90 therapy is gastrointestinal ulceration caused by extrahepatic dispersion of microspheres. Standard pretreatment planning requires extensive angiographic evaluation of the hepatic circulation and embolization of hepatoenteric collaterals; however, in patients with severe renal insufficiency, this evaluation may lead to acute renal failure. In order to minimize iodinated contrast utilization in a patient with preexisting severe renal insufficiency, the authors describe the use of a balloon catheter for temporary occlusion of the common hepatic artery to induce transient redirection of flow of the hepatoenteric arteries towards the liver, in lieu of conventional coil embolization.

## 1. Introduction

The use of yttrium-90 radioembolotherapy of the liver has become increasingly more widespread. Indications include palliative therapy for unresectable metastatic liver disease and bridging therapy to liver transplant in patients with primary liver malignancy [1]. The conventional approach to yttrium-90 microsphere radioembolotherapy involves prior angiographic identification of the hepatoenteric arteries with subsequent prophylactic coil embolization of these vessels to prevent radiation-induced gastrointestinal ulceration, gastrointestinal bleeding, or pancreatitis [2]. This involves the additional administration of significant amounts (up to 150 mL) of iodinated contrast to monitor, modify, and confirm the occlusion process [3]. Unfortunately, patients with chronic kidney disease (CKD) may be excluded from this therapy given the risk of contrast-induced nephropathy (CIN). While ample pre- and postprocedural hydration remains the mainstay for prevention, the absolute volume of contrast that is administered has been shown as an independent predictor of CIN [4]. Temporary balloon occlusion of the common hepatic artery during yttrium-90 glass or resin microsphere therapy to induce transient hepatopetal flow in the hepatoenteric arteries has been described [5, 6]. This technique obviates the need for prophylactic coil embolization of the hepatoenteric arteries and may minimize additional iodinated contrast exposure. The experience of this technique in patients with compromised renal function has not been reported. We currently describe the safety and efficacy of temporary balloon occlusion for yttrium-90 radioembolotherapy in a patient with advanced chronic kidney disease.

## 2. Case Report

HIPAA approval for a single patient report is waived by our institutional review board. A 68-year-old Caucasian male with history of hypertension, diabetes, and CKD was initially diagnosed with a 5 cm left (segment II/III) lobe hepatocellular carcinoma (HCC) that was resected at an outside institution. Bilobar hypervascular liver lesions consistent with multifocal HCC were present on six month follow-up imaging. After evaluation at our multidisciplinary liver tumor board, a recommendation was made for combination therapy with systemic sorafenib (Onyx Pharmaceuticals, San Francisco, CA, and Bayer Pharmaceuticals, Leverkusen, Germany) and liver-directed TheraSphere yttrium-90 radioactive glass microspheres (MDS Nordion, Ottawa, ON, Canada). The patient was initiated on sorafenib 400 mg PO BID and experienced a deterioration in his creatinine from a baseline of 1.85 mg/dL to 2.44 mg/dL. Since angiogenesis inhibitors such as sorafenib are known to cause acute kidney injury and proteinuria, the dose was decreased to 200 mg PO BID.

**Figure 1:**
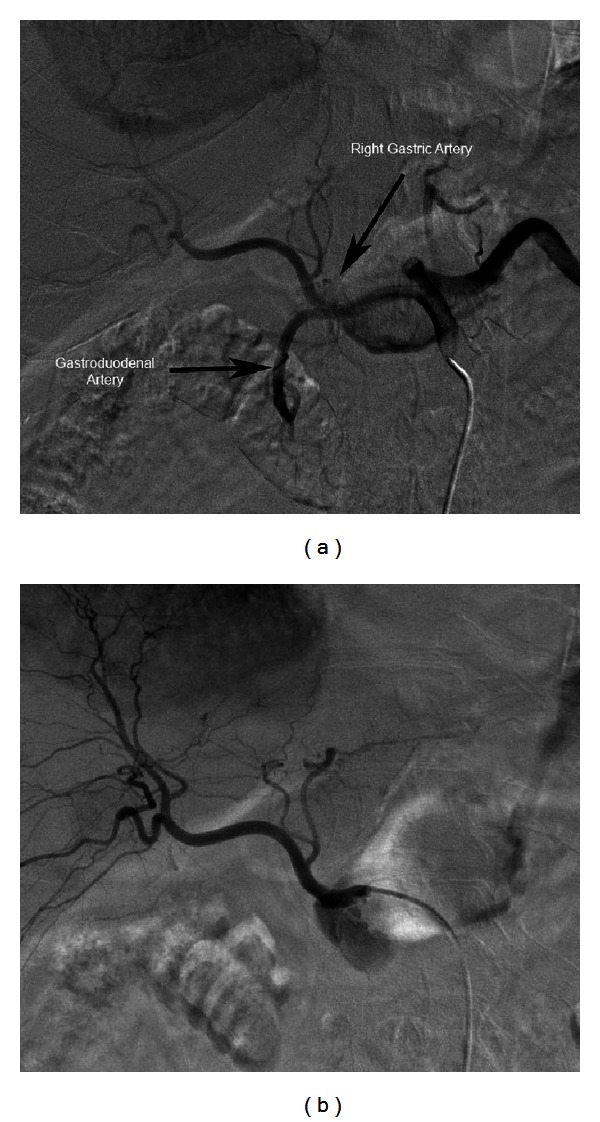
(a) Celiac angiogram demonstrates antegrade flow in the right gastric and gastroduodenal arteries. (b) Common hepatic angiogram with balloon inflation demonstrates absence of antegrade flow in the right gastric and gastroduodenal arteries.

**Figure 2:**
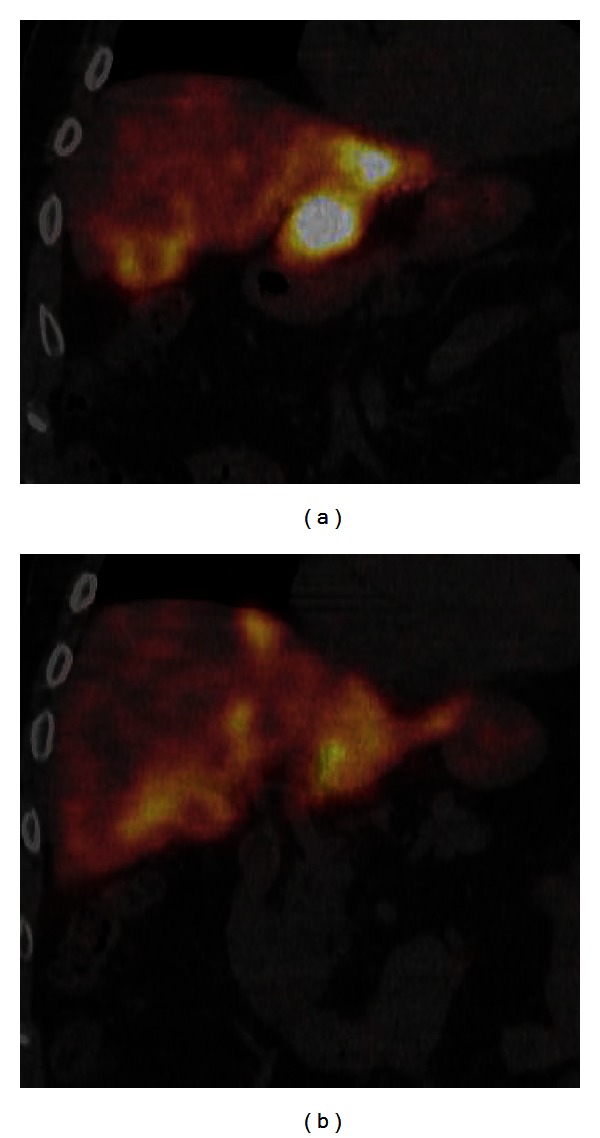
(a) and (b) Coronal fused SPECT/CT MAA study demonstrates multifocal hepatic uptake without gastrointestinal activity.

We proceeded with therapy planning arteriography with the intent to minimize iodinated contrast use. Celiac axis (Figure 1(a)) and superior mesenteric digital subtraction arteriography (DSA) were performed demonstrating type I, standard celiac axis arterial anatomy. A 5.5 French over-the-wire Fogarty balloon catheter (Edwards Life Sciences, Irvine, CA) was placed in the common hepatic artery (CHA) under fluoroscopy proximal to the origin of the gastroduodenal artery (GDA). After balloon inflation, a CHA DSA demonstrated the absence of enteric flow in the GDA and right gastric artery (RGA) (Figure 1(b)). Additional extrahepatic supply was absent. Also, 5 mCi of Technetium 99 m macroaggregated albumin (MAA) was then administered prior to deflating the balloon. Radioactivity was confined to the multiple hepatic lesions with no gastrointestinal activity on scintigraphy (Figures 2(a) and 2(b)). A total of 27 mL of Visipaque iodinated contrast (GE Healthcare, Princeton, NJ) was used. Serum creatinine the following day measured 2.32 mg/dL, which was stable from preprocedure values.

Upon return for yttrium-90 radioembolotherapy two weeks later, the serum creatinine was 2.24 mg/dL. On the day of treatment, celiac arteriography was performed using identical technique as for the therapy planning after the intravenous administration of 4000 units of unfractionated heparin to prevent CHA thrombosis. Absence of enteric flow via the GDA and RGA was reconfirmed. A Renegade microcatheter (Boston Scientific, Natick, MA) was placed coaxially into the proper hepatic artery (PHA), and the radioactive microspheres were delivered. The injection rate of 3.51 GBq TheraSpheres was manually regulated to mimic the flow rate of the PHA DSA that confirmed the lack of enteric flow [2]. A postmicrosphere delivery CHA arteriogram revealed no changes in the flow characteristics. A total of 34 mL of Visipaque contrast was used. A posttherapy serum creatinine was not measured due to out-of-state travel conflicts.

A decrease in renal function secondary to urinary tract infection and reinitiation of sorafenib was documented nearly 3 months later with a serum creatinine of 3.45 mg/dL (GFR 19 mL/min/1.73 m^2^). Follow-up MRI 5 months after yttrium-90 radioembolotherapy showed a 50% decrease in liver tumor burden, classified as partial response by World Health Organization (WHO) criteria. There were no gastrointestinal adverse events that could be ascribed to radioembolotherapy, specifically no episodes of abdominal pain, gastrointestinal bleeding, or unexplained anemia.

## 3. Discussion

In this case, we demonstrated the successful use of temporary balloon occlusion of the common hepatic artery for the delivery of yttrium-90 glass microspheres in a patient with advanced chronic kidney disease. We were able to limit the amount of iodinated contrast necessary compared to conventional therapy and therefore avoid CIN. Furthermore, the patient demonstrated a 50% decrease in liver tumor burden by WHO criteria, supporting the efficacy of our therapy.

The need for significant amounts of IV iodinated contrast may preclude conventional radioembolotherapy in patients with advanced CKD given the risk of CIN. Our patient had advanced chronic kidney disease secondary to hypertension and diabetes with a baseline serum creatinine of 1.8 mg/dL prior to the initiation of anti-VEGF therapy (sorafenib). His renal function gradually deteriorated over the next 7 months on sorafenib; however, there was no evidence of acute kidney injury from contrast exposure after our therapy. The creatinine remained stable prior to therapy planning arteriography, immediately afterwards and 2 weeks later. When present, CIN typically occurs within 24–48 hours of exposure with serum creatinine levels peaking in 3–5 days [7]. Furthermore, it has been shown that appreciable nephropathy is unlikely to develop if the serum creatinine level does not increase by more than 0.5 mg/dL within 24 hours [8]. Ideally, we would have obtained a serum creatinine immediately after the second contrast exposure during radioembolotherapy. However, the amount of contrast given was similar to the initial contrast exposure 2 weeks prior (34 mL versus 27 mL), which was well tolerated by the patient. Therefore, we strongly feel that it was unlikely that CIN was not a significant contributor to the decline in renal function. The balloon occlusion technique has been described with success in chemoembolization [9], as well as in radioembolization [5, 6]. Adoption of this technique allowed us to safely administer glass radiomicrospheres in the presence of patent hepatoenteric arteries without the addition of contrast required for hepatoenteric artery embolization. This balloon occlusion technique does have limitations as it has only been applied in patients with type I standard celiac arterial anatomy. Additionally, because of the relative inflexibility of currently available balloon catheters, arterial spasm or dissection can occur. Tortuosity of the common hepatic artery may also pose technical difficulties that can preclude adequate positioning. In our case, none of these limitations were present.

In summary, we have demonstrated a safe and effective method for the delivery of yttrium-90 microspheres in a patient who would not have qualified for conventional radioembolotherapy at our institution secondary to advanced CKD.
